# The Characteristics of the Metal-Free and Non-Conjugated Polymer Film with Self-Assembled Nanoparticles

**DOI:** 10.3390/nano13030596

**Published:** 2023-02-02

**Authors:** Kwang-Ming Lee, Chung-Cheng Chang, Jia-Ming Wang, Chia-Yu Chang, Chia-Hong Huang

**Affiliations:** 1 Department of Chemistry, National Kaohsiung Normal University, Kaohsiung 824004, Taiwan; 2 Department of Electrical Engineering, National Taiwan Ocean University, Keelung 202301, Taiwan; 3 Department of Electronic Engineering, National Kaohsiung Normal University, Kaohsiung 824004, Taiwan

**Keywords:** antireflection, self-assembled nanoparticles, optical polymer film, host

## Abstract

It is shown in this paper that a polymer, MA-PEG 1000-DGEBA (MP1D), exhibits antireflection, substrate-dependent photoluminescence (SDP), wide band-gap, and photoconduction characterization. MP1D was synthesized from maleic anhydride, polyethylene glycol 1000, and bisphenol-A diglycidyl ether. Self-assembled nanoparticles embedded in MP1D film and ranging from 2.5 to 31.6 nm are observed, which could be expected as scatterers to enhance light trapping and extraction. The size of the nanoparticle increases with the concentration of the MP1D solution. Besides solution concentration, the nanoparticle dimension could be modified by the chain length of polyethylene glycol in the polymer synthesis. The effects of solution concentration, annealing temperature, annealing period, and substrate on the photoluminescence (PL) of MP1D films are examined. Increasing solution concentration increases PL intensity. However, aggregation-caused quenching is explicit as the solution concentration exceeds 100 mM. PL intensity increases with annealing temperature, which could be attributed to crystallinity improvement. PL intensity increases with increasing the annealing period from 0.5 to 2 h. Nonetheless, as the annealing period exceeds 2 h, PL quenching is emerging, which could be due to aggregation. It is expected that MP1D could be a promising candidate for host materials and MP1D film could play a multifunctional role (antireflective and light-trapping functions) in optoelectronics.

## 1. Introduction

Optical thin films provide significant performance enhancements in optical and optoelectronic applications. Films such as anti-reflective (AR) films, anti-glare films, light-control films, brightness-enhancement films, and device-protective films coated on components or devices can increase light transmittance, diffuse mirror-like reflections, redirect light propagation, enhance optical properties, extend lifetime or improve image quality. Because of the difference in the refractive index between air, films, and substrate, Fresnel reflection occurs as light enters or leaves the surface of optical or optoelectronic devices so as to affect the efficiency of light incoupling or outcoupling. So, light reflection loss degrades the performance of optical and optoelectronic devices. For instance, owing to total internal reflection and evanescent coupling, organic light-emitting diodes (OLEDs) usually exhibit approximately 20% external quantum efficiency (EQE) without using light extraction technologies [1,2]. Additionally, the surface of a polymer bulk heterojunction photovoltaic device reflects around 30% of the incident light without anti-reflective (AR) structures [3]. Therefore, the optical film which can decrease light reflection and increase light transmission is a key element to improve the performance of optoelectronic devices, so AR films are paid much attention. Traditionally, the composition of the optical film is single- or multiple-layered depending on the intended purposes and required performance. The single-layer film is facile but cannot lower reflection in a broadband spectrum [4]. Conversely, the multilayer film is complex to manufacture because of the demand for an appropriate refractive index and accurate thickness. Moreover, thermal-expansion mismatch and material migration in the multilayer film destroys its structure under a high power condition [5]. Fortunately, an alternative to single- and multiple-layered films is a nanostructured film. Nanostructured films with a size of nanostructure comparable to the wavelength of light attract intensive interest because of their unconventional features, such as scattering properties of random structures embedded in the films and plasmonics excited in metallic nanoparticles or at metal/dielectric interfaces. Compared with periodic nanostructures, disorder nanostructures enable avoiding the angular dependence on light wavelength. In past decades, nanostructured films have been used to reduce reflection and enhance device performance in photovoltaic (PV) [6,7,8,9,10,11], photodetectors [12], displays [13,14], light-emitting diodes [9,15,16,17,18,19], and laser [20] applications.

Nanostructured films, whether inorganic or organic, are manufactured by various technologies that generally involve imprinting [7], molding [9], etching [16], photolithography [14], or vacuum processing [15]. Unfortunately, these techniques are relatively complicated or expensive for low-cost, large-area, and high-volume fabrication. Thus, there has been considerable attention in wet processing because of the simple and cost-efficient methods. Furthermore, recent studies have demonstrated that nanoparticles embedded in optical films can improve light diffusion, extraction, and transmission in optoelectronic devices [11,17,21,22,23]. Importantly, optical films with nanoparticles are industrially promising candidates that could enhance light extraction efficiency and be cost-efficient in OLED applications [1]. In the previous study, a non-conjugated polymer was synthesized without utilizing organometallic reagents and solvents [24]. Self-assembled nanoparticles embedded in the polymer films were found. It has been suggested that self-assembled nanoparticles are generated with bisphenol-A aggregates and poly(ethylene glycol) moieties in the synthesis process [24]. Self-assembly is a cost-effective and high-yield process to build nano-scale structures. In this study, the MP1D polymer was manufactured in an eco-friendly process without employing solvents. In addition, MP1D film with self-assembled nanoparticles shows AR and substrate-dependent photoluminescence (SDP) characteristics. Especially, the major benefit of such random nanoparticles is that the spectra of reflection and transmission are not dependent on view angle and incident light wavelength. Consequently, MP1D film could be expected as a scattering film to enhance light trapping and extraction in optoelectronic applications. Besides, traditional conjugated polymers have not only severe environmental concerns but also involve aggregation-caused quenching (ACQ) [25]. Hence they are considerably limited in solid-state or optoelectronics. Conversely, the luminescence of MP1D film is not obviously quenched up to 100 mM in this study. Furthermore, a slightly red shift (6 nm) of the PL spectrum is found as solution concentration increases. Similarly, a rise in PL peak intensity is accompanied by a slightly red shift (8 nm) in PL spectra when the annealing temperature increases. In addition, PL intensity rises with the annealing period. However, as the annealing period is more than 2 h, PL quenching is observed. Moreover, UV-Vis absorption spectra of MP1D films present a red shift with increasing solution concentration, which is due to more molecular aggregation and stronger intermolecular interactions. The optical band-gap energy (*E*_g_) of MP1D film is around 4.05 eV. Accordingly, it is expected that MP1D polymer could not only be utilized as a host material but could also be applied to antireflection owing to its wide band-gap and low reflective properties for multifunctional optoelectronic applications.

## 2. Materials and Methods

### 2.1. Synthesis of MP1D Polymer

MP1D polymer was synthesized using the process described previously [24]. All chemicals were utilized as received without any processing. Initially, 0.1 g of maleic anhydride and 0.5 g of polyethylene glycol 1000 were mixed and then stirred under N_2_ at 90 °C for 1.5 h without utilizing any solvent. The product was recrystallized twice from ether. Afterward, the mixture of the product (5.5 g) and bisphenol A diglycidyl ether (1.57 g) was stirred at 120 °C for 3 h in N_2_, and then returned to room temperature. Eventually, a light brown product was obtained and then called MA-PEG 1000-DGEBA (MP1D).

### 2.2. Preparation of MP1D Film

MP1D polymer was added into tetrahydrofuran (THF). MP1D films produced by using MP1D solution were spin-coated for 60 s with 2000 rpm on ITO-coated glass and Si substrates, followed by annealing at 120 °C in N_2_ for 1 h. Furthermore, to examine the effect of ACQ on the luminescence of MP1D film, MP1D films produced from different solutions (50, 75, 100, 125, and 150 mM) were manufactured on ITO-coated glasses. Besides, to investigate the influence of annealing temperatures and periods on PL of MP1D films, the films were annealed at different temperatures (80, 90, 100, 120, 150, and 200 °C) and for various periods (0.5, 1, 2, and 4 h).

### 2.3. Preparation of ITO/MP1D/Al Sandwich-Type Devices

The ITO-coated glass slides with a sheet resistance of 8–12 Ω/sq were cleaned in chloroform, acetone, and de-ionized (DI) water for 10 min each, respectively. The area of the ITO-coated glass slide was 1.5 × 1.5 cm^2^. MP1D solution was spin-coated onto ITO-coated slides at 2000 rpm for 60 s, followed by annealing on a hot plate at 120 °C for 1 h. An Al film was then deposited on MP1D film for all samples by thermal evaporation. Finally, all samples were annealed in a furnace at 100 °C in N_2_ for 10 min. Al electrode area was 1.5 × 1.5 mm^2^.

### 2.4. Characterization

The thicknesses of MP1D films deposited by using various solutions (50, 75, 100, 125, and 150 mM) were estimated by surface profiler (Bruker Dektak XT, Billerica, MA, USA) and were 67, 70, 77, 132, and 267 nm, respectively. The current-voltage curves of the ITO/MP1D/Al sandwich-type devices were measured by Keitheley 4200 semiconductor analyzer. The photocurrent was obtained in AM 1.5 G solar spectrum with a power density of 100 mW/cm^2^ at 25 °C. To analyze the elemental composition of MP1D film, X-ray photoelectron spectroscopy (XPS) was measured by an X-ray photoelectron spectrometer (ULVAC-PHI, PHI Quantera II, Kanagawa, Japan) equipped with a monochromatic Al Kα X-ray source (1486.6 eV). To observe self-assembled nanoparticles, tunneling electron microscope (TEM) images were employed (JEOL JEM-2100 TEM, Tokyo, Japan). PL characteristics were performed on a FluoroMax-4 PL spectrometer (Horiba Jobin Yvon, Kyoto, Japan) under illumination of 325 nm wavelength at room temperature. Ultraviolet-visible (UV-Vis) absorption and reflectance spectra were conducted by using an UV/VIS/NIR spectrophotometer (UV-3150, Shimadzu Corporation, Tokyo, Japan). The optical band-gap energy (*E*_g_) was measured from UV-Vis absorption spectra.

## 3. Results and Discussion

The elemental composition of MP1D film was examined by XPS, as shown in Figure S1. It is obvious that C 1s and O 1s peaks are in the XPS spectrum. Moreover, the XPS survey demonstrates that only carbon and oxygen exist in MP1D film. Therefore, it indicates that MP1D film is metal-free. Furthermore, the chemical structure of the MP1D polymer was characterized by ^1^H nuclear magnetic resonance (NMR) and Infrared (IR) spectrum, as illustrated in Figures S2 and S3, respectively.

**Figure 1:**
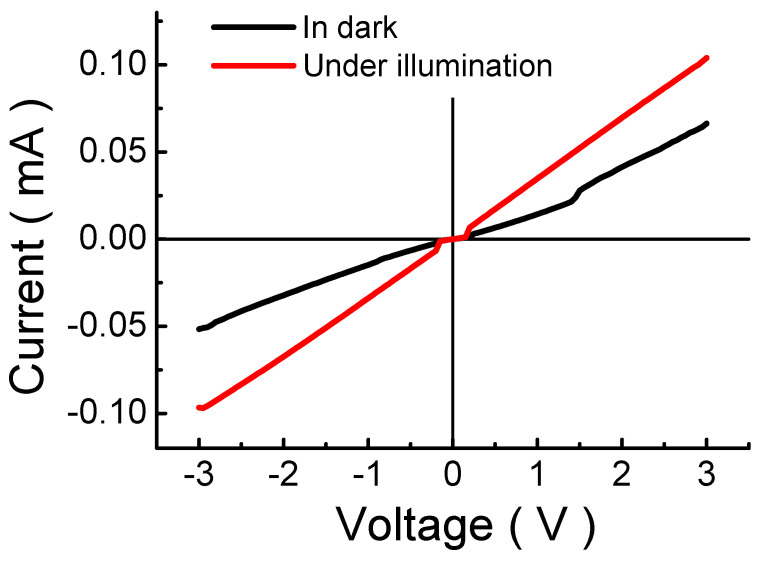
Current-voltage (I-V) curves of MP1D film in dark and under illumination.

To examine the conductivity of MP1D polymer, we prepared ITO/MP1D/Al sandwich-type devices in which the thickness of MP1D film was about 77 nm. The conductivity was measured from −3 to 3 V. Figure 1 presents current-voltage (I-V) properties of MP1D film in the dark and under illumination. It is clear that all curves are symmetric about zero and linear. MP1D film possesses a resistivity of approximately 1.12 × 10^6^ Ω·m in the dark so it is an intrinsic semiconductor. The conductivity increases from its dark value of 8.9 × 10^−7^ S·m^−1^, reaching 1.2 × 10^−6^ S·m^−1^ under the illumination of 1000 W·m^−2^. The light illumination increases the conductivity of MP1D film by only 1.2 times, which can be attributed to the shorter absorption cut-off wavelength (around 306 nm estimated from Figure S4). Thus MP1D polymer has a wide optical band-gap energy (*E*_g_) of approximately 4.05 eV. The wavelength of 306 nm lies in the ultraviolet (UV) region. The spectral irradiance of UV light is much less than that of visible light in AM 1.5 G spectrum. Therefore, the photogenerated electrons in MP1D film are not huge under AM 1.5 G illumination, so the increase in conductivity is not apparent. In Figure S4, it is obvious that the UV-Vis absorption spectra of MP1D films shift to longer wavelengths (red shift) with increasing solution concentration. This result implies that more molecular aggregation and stronger intermolecular interactions exist in polymer films produced with higher concentrated solutions [26]. It is proposed that more molecular aggregation causes the larger size of the cluster with the higher concentration solutions and thus reduces *E*_g_ based on the quantum size effect [27].

**Figure 2:**
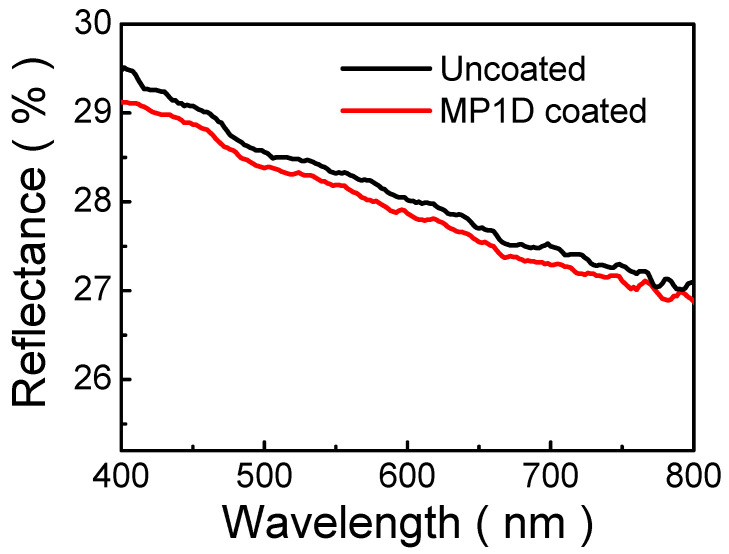
Reflectance spectra of MP1D film on ITO glass.

To investigate the reflectance of MP1D film, it was spin-coated on an ITO-coated glass (i.e., an MP1D-coated sample). Figure 2 depicts the reflectance spectra of the MP1D film. The reflectance of an MP1D-coated sample is small in comparison with that of the ITO-coated glass (i.e., an uncoated sample) in a wavelength range of 400–800 nm, as presented in Figure 2. It obviously demonstrates that MP1D film has AR characteristics. Moreover, the reflectance of an MP1D-coated sample slightly decreases with wavelength. This is because the step gradient refractive-index distribution is in multiple films. In the case of a single-layered AR film, a single coating is deposited on the device surface, so that light reflections from the air/AR film and AR film/device surface interfaces undergo interference. To eliminate reflection, this interference has to be a destructive interference. As a consequence, the thickness of a single-layered AR film must be odd-multiples of the quarter wavelength in the coating and thus depends on the wavelength. Moreover, the refractive index of a single-layer AR film equals the square root of the refractive index of the device surface. MP1D film thickness is around 77 nm and is not optimized in Figure 2. Hence, the reflectance is not greatly reduced.

**Figure 3:**
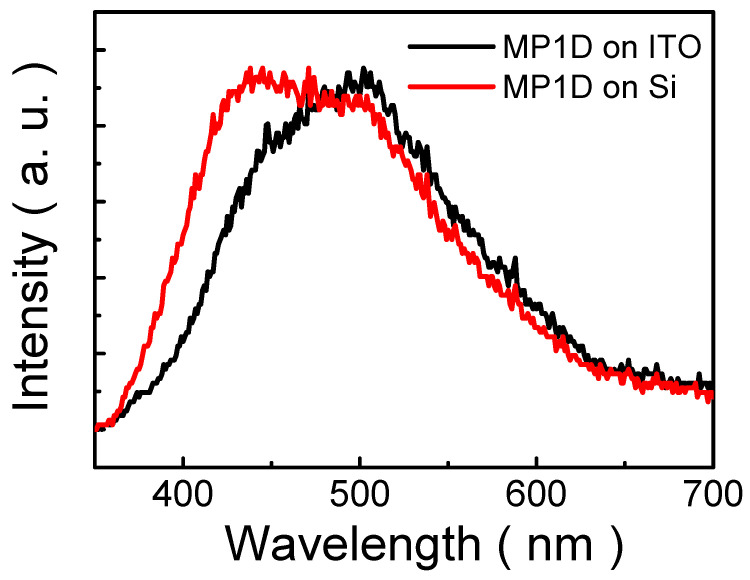
Room temperature PL spectra of MP1D films on ITO and Si substrates.

Figure 3 illustrates PL spectra of MP1D films coated on Si and ITO glass substrates under the illumination of 325 nm wavelength. The wavelengths of PL peaks of MP1D films on Si and ITO are 471 and 494 nm, respectively. PL spectrum of MP1D film on ITO exhibits a red shift (23 nm) compared with that on Si. In the literature, PL in nanostructures is intensively influenced by strain [28,29,30,31,32], doping [33,34,35,36,37], and dielectric screening [38]. The lattice constants of Si and ITO are 5.43 Å and 10.12 Å, respectively. Therefore, the induced strain of MP1D film on Si is more than on ITO, which is due to a comparatively larger lattice mismatch between MP1D and Si. Furthermore, if the effect of the lattice mismatch-induced strain is dominant in PL, a red shift of the PL spectra would be found as the strain increases [28,29,30,31,32]. Nevertheless, it is in opposition to our results in Figure 3. In addition, thermal annealing is at 120 °C in production processing. Thus, the influence of doping on luminescence is negligible in this study. Besides, it has also been reported that the influence of the dielectric environmental screening on the PL of nanostructures is significant [38]. From Figure 3, it is found that increasing the relative dielectric constant of the underlying film or substrate, from 3.4 (ITO) to 11.7 (Si), decreases the PL peak wavelength (a blue shift). It could be attributed to the dielectric screening of Coulomb interactions, which can influence the exciton binding energy [38]. That is, the larger relative dielectric constant of the underlying film or substrate causes the smaller Coulomb interaction between electron and hole so it lowers the exciton binding energy [39,40]. As a result, the energy of the emission photon increases. Accordingly, if the influence of dielectric screening is dominant in the PL process, a blue shift is observed in the PL spectra as the relative dielectric constant of the neighboring dielectrics increases. It is because the PL peak energy can be evaluated by subtracting the exciton binding energy from the band-gap energy, which is consistent with our results. Therefore, it is proposed that a blue shift of the PL spectra of MP1D film with neighboring dielectrics is attributed to dielectric screening.

**Figure 4:**
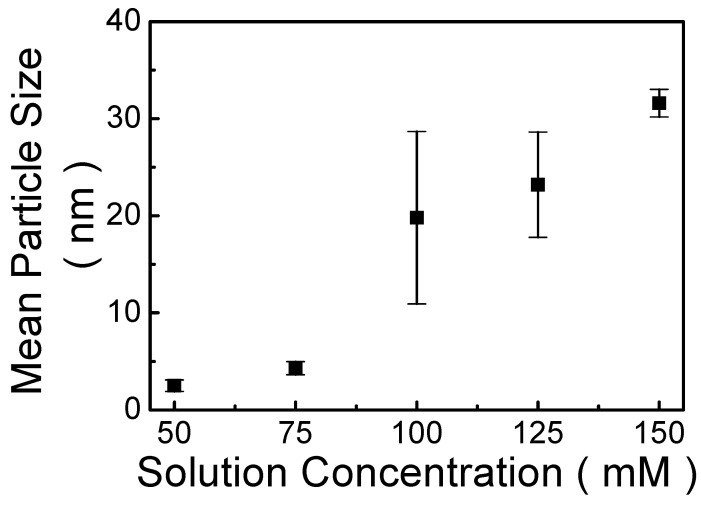
The mean size of the self-assembled nanoparticle as a function of solution concentration.

The TEM images in Figure S5, show that the nanoparticles embedded in MP1D film are dispersive. Simultaneously, the size of the nanoparticle is not uniform. The size distribution of the self-assembled nanoparticles is fitted by a Gaussian curve, affirming the average size (μ ± σ) at 2.5 ± 0.59, 4.3 ± 0.67, 19.8 ± 8.88, 23.2 ± 5.42 and 31.6 ± 1.42 nm for MP1D films prepared from 50, 75, 100, 125 and 150 mM of MP1D solutions, respectively. Figure 4 presents that the mean size of the self-assembled nanoparticle varies at various concentrations. It is found the mean size of the self-assembled nanoparticles increases with the solution concentration. Generally, to construct nanostructures, the self-assembly of organic molecules occurs in solution. Consequently, it has been suggested that the self-assembled nanoparticle is formed from bisphenol-A aggregates and polyethylene glycol moieties serving as core and shell compartments, respectively [24]. Accordingly, the higher concentrated solution provides a larger amount of bisphenol-A aggregates and poly(ethylene glycol) moieties, thus the dimension of the nanoparticle enlarges with the solution concentration. In other words, increasing the concentration of the MP1D solution increases the size of the self-assembled nanoparticle embedded in the MP1D film. This trend is the same in the literature [41]. Nonetheless, under the same condition of 50 mM solution, the size of the nanoparticle embedded in MP1D film is much smaller than that (52.8 nm) of the nanoparticle embedded in the A-PEGCP film reported in the literature [41]. It is because the polymer chain of polyethylene glycol 1000 used in the synthesis of MP1D polymer is shorter than that of polyethylene glycol 6000 used in the synthesis of A-PEGCP polymer so that poly(ethylene glycol) moieties in MP1D film are smaller than those in A-PEGCP film. Thus, the size of the nanoparticle embedded in the MP1D film is smaller than that of the nanoparticle embedded in the A-PEGCP film. Hence, in addition to the solution concentration, the nanoparticle size can be altered by the chain length of polyethylene glycol in the polymer synthesis.

Furthermore, nanoparticles embedded in films can scatter light in many directions [22]. Consequently, scattering is more effective than absorption for light harvesting in solar cells [42]. It has also been shown that the larger nanoparticle leads to more scattering [22]. Nevertheless, larger nanoparticles embedded in the film inevitably cause an increase in the film thickness. As a consequence, the absorption in the film rises simultaneously. Today, nanoparticles have been further studied, and are classified into metallic and dielectric nanoparticles. Even though metallic nanoparticles intensively scatter light at the wavelength of the incident light near their resonant wavelength, their losses are critical. Most importantly, the resonant scattering of the metallic nanoparticle only arises from the electric resonances, while dielectric nanoparticles possess both electric and magnetic resonances synchronously excited by an incident light [43]. As a result, dielectric nanoparticles could be more efficient for light scattering and trapping for optoelectronic applications.

**Figure 5:**
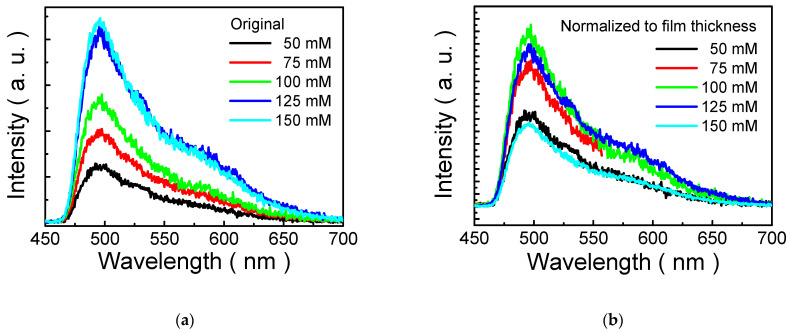
(**a**) PL spectra (original) of MP1D films coated from different solutions (50, 75, 100, 125, and 150 mM); (**b**) PL spectra (normalized to film thickness) of MP1D films coated from 50, 75, 100, 125, and 150 mM solutions.

Figure 5a displays the PL spectra of MP1D films spin-coated from various solutions (50, 75, 100, 125, and 150 mM). The wavelength of the PL peak is slightly varied from 493 to 499 nm as solution concentration increases from 50 to 150 mM. Namely, a slight red shift in PL spectra is found while the concentration increases. In general, it was suggested that a red shift in PL spectra with increasing the dimension of the nanostructure is due to the quantum size effect [27]. Accordingly, we proposed that increasing the solution concentration increases the nanoparticle size in this study. This could be ascribed to the larger amount of MP1D polymer in a higher concentrated solution, which results in more aggregation of bisphenol-A aggregates and polyethylene glycol moieties, and thus the dimension of the nanoparticle enlarges. It is in good agreement with the observation of Figure 4.

Moreover, it also reveals that the PL peak intensity of MP1D film is concentration-dependent. Increasing solution concentration increases PL peak intensity, as shown in Figure 5a. Conventional fluorescent conjugated materials usually possess strong emission characteristics in diluted solutions, whereas luminescence is quenched significantly in concentrated solutions or in a solid state. It is with regard to aggregation-caused quenching (ACQ) [25]. In Figure 5a, ACQ is not obvious in MP1D films.

Incidentally, it is also believed that PL intensity is thickness-dependent. To investigate the dependence of the luminescence of MP1D film on solution concentration in detail, PL intensity is normalized to film thickness to eliminate the effect of film thickness, so that the normalized PL spectra are independent results in Figure 5b. PL quenching is not observed up to 100 mM. However, a quench in PL spectra is clear at higher concentrations (≥125 mM), as presented in Figure 5b. In comparison, a downward movement in PL peak intensity of MP8B film occurs at 10 mM solution in our previous study [24]. This is because the polymer chain of polyethylene glycol 1000 used in the synthesis of MP1D polymer is shorter than that of polyethylene glycol 8000 used in the synthesis of MP8B polymer. Therefore, it is proposed that MP8B polymer can pack closer together than MP1D polymer so that the degree of ACQ in MP8B film is more serious than that in MP1D film.

**Figure 6:**
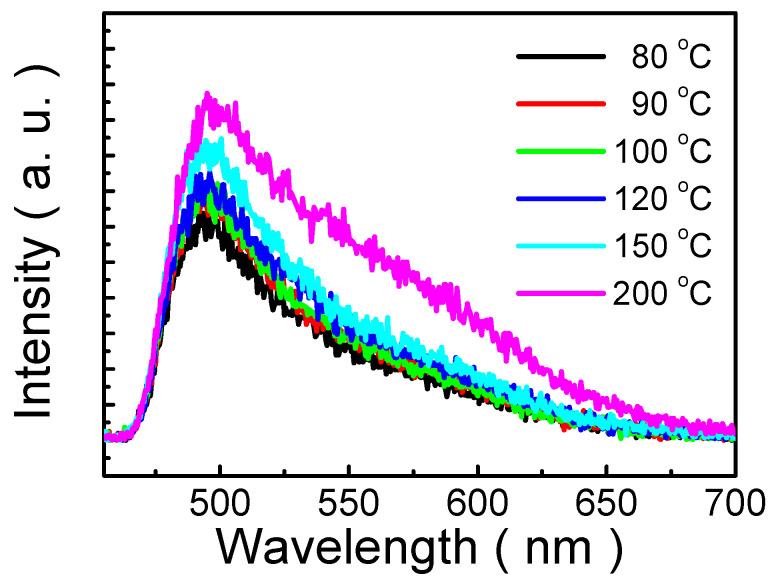
PL spectra of MP1D films annealed at various temperatures (80, 90, 100, 120, 150, and 200 °C).

Figure 6 shows PL spectra of MP1D films annealed at different temperatures (80, 90, 100, 120, 150, and 200 °C). An increase in the intensity of the PL peak is accompanied by a slightly red shift (8 nm) in PL spectra as the annealing temperature rises from 80 °C to 200 °C. PL peak intensity increases with annealing temperature, which could be attributed to crystallinity improvement. It is deduced that during thermal annealing, solvent evaporation leads to molecular aggregation and subsequent reconstruction of the grains. Consequently, the higher the annealing temperature, the bigger the grain size, so the PL peak intensity rises with the annealing temperature. Moreover, the main peak is at approximately 496 nm, which is due to intrachain exciton emissions. On the other hand, indistinct shoulders at around 542 and 580 nm are observed in the PL spectrum of 200 °C annealing temperature, which could be ascribed to emissions from aggregates of interchain species [44]. In general, the shoulder intensity increases with the annealing temperature, suggesting that the density of the interchain species increases after higher annealing temperature [45]. Accordingly, from the result of Figure 6, it is proposed that the density of the interchain species in MP1D film is not significant until 150 °C of annealing temperature, while, at the higher annealing temperature (>150 °C), the density of the interchain species would slightly increase.

**Figure 7:**
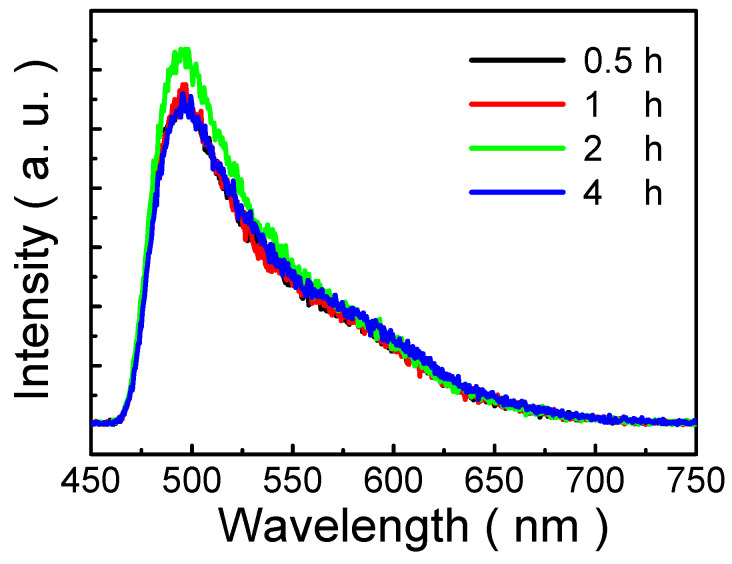
PL spectra of MP1D films annealed for various periods (0.5, 1, 2, and 4 h).

Figure 7 shows the PL spectra of MP1D films annealed for various periods (0.5, 1, 2, and 4 h). The shape of the PL spectra of MP1D films is asymmetric. It has been suggested that the asymmetric shape in the PL spectrum implies the presence of localized (zero-dimensional) states [46]. The localized states are in the vicinity of the band edges. In Figure 7, it is clear that the PL peak intensity increases with increasing annealing period (from 0.5 h to 2 h) and shows a maximum of the PL peak intensity for 2 h and after that, it decreases. In the literature, it was shown that the morphology of conjugated polymer abruptly changes at the initial stage of the annealing period [47]. Accordingly, it is suggested that the morphology of MP1D film rapidly varies at the shorter period (≤1 h), and is then stable up to 2 h. Nonetheless, as the annealing period is 4 h, the degree of aggregation rises, so that PL quenching is emerging, as illustrated in Figure 7. Moreover, because of the absence of interchain interactions in the MP1D film, the PL characteristics of MP1D films are similar except for the PL peak intensity for various annealing periods in Figure 7. Therefore, it is important to establish the optimum processing conditions (the period and temperature of thermal annealing) for the morphology of MP1D film in optoelectronic applications.

## 4. Conclusions

MP1D is a wide band-gap, metal-free, and non-conjugated polymer, which is an intrinsic semiconductor. Moreover, MP1D film prepared from MP1D solution can decrease reflectance. The PL peak of MP1D films on ITO exhibits a red shift (23 nm) compared with that on Si. Such SDP is ascribed to dielectric screening. From TEM images, the mean size of self-assembled nanoparticles embedded in MP1D films becomes larger (from 2.5 to 31.6 nm) with the solution concentration, which is attributed to the more self-assembled formation of bisphenol-A aggregates and poly(ethylene glycol) moieties in the higher concentration solution. In addition to solution concentration, the nanoparticle size could be altered by changing the chain length of polyethylene glycol in the polymer synthesis. Self-assembled nanoparticles embedded in MP1D film are expected as scatterers to enhance light trapping and extraction in light harvesting and source applications. Furthermore, the PL peak illustrates a slightly red shift (6 nm) with increasing solution concentration, from 50 to 150 mM, because of the quantum confinement effect. It is consistent with the result of TEM images. Besides, PL peak intensity is dependent on solution concentration. PL quenching is not observed up to 100 mM, eliminating the effect of film thickness. Nonetheless, when the solution concentration is more than 100 mM, there is a descending tendency in PL peak intensity. In addition, an increase in PL peak intensity is accompanied by a slightly red shift (8 nm) in PL spectra as the annealing temperature rises from 80 °C to 200 °C. Additionally, PL peak intensity increases initially with an increasing annealing period from 0.5 to 2 h. Nevertheless, as the annealing period exceeds 2 h, PL peak intensity decreases, which could be due to aggregation. Importantly, the morphology of MP1D film can be varied by changing the annealing temperature and period. The optical band-gap energy is around 4.05 eV. From the above results, it is expected that MP1D could play an important role in host materials and MP1D film with self-assembled nanoparticles could become a multifunctional film (antireflective and light-trapping functions) for optoelectronic applications.

## Data Availability

The data presented in this study are available on request from the corresponding author.

## Supplementary Materials

The following are available online at https://www.mdpi.com/article/10.3390/nano13030596/s1, Figure S1: XPS spectrum of MP1D film was recorded on an X-ray photoelectron spectrometer; Figure S2: Chemical structure and the ^1^H NMR (400 MHz, CDCl_3_) spectrum of MP1D polymer; Figure S3: IR spectrum of MP1D polymer; Figure S4: UV-Vis absorption spectra of MP1D films coated from various solutions (50, 75, 100, 125 and 150 mM); Figure S5: The TEM images of MP1D films prepared from different MP1D solutions: (a) 50 mM; (c) 75 mM; (e) 100 mM; (g) 125 mM; (i) 150 mM. The histogram of the size distribution of self-assembled nanoparticles embedded in MP1D films prepared from different MP1D solutions: (b) 50 mM; (d) 75 mM; (f) 100 mM; (h) 125 mM; (j) 150 mM.

## References

[1] Gomard G., Preinfalk J.B., Egel A., Lemmer U. (2016). Photon management in solution-processed organic light-emitting diodes: A review of light outcoupling micro- and nanostructures. J. Photonics Energy.

[2] Gu G., Garbuzov D.Z., Burrows P.E., Venkatesh S., Forrest S.R., Thompson M.E. (1997). High-external-quantum-efficiency organic light-emitting devices. Opt. Lett..

[3] Park S.H., Roy A., Beaupré S., Cho S., Coates N., Moon J.S., Moses D., Leclerc M., Lee K., Heeger A.J. (2009). Bulk heterojunction solar cells with internal quantum efficiency approaching 100%. Nat. Photonics.

[4] Zheo J., Wang A., Atermatt P., Green M.A. (1995). Twenty-four percent efficient Silicon Solar Cells with Double Layer Antireflection Coatings and Reduced Resistance Loss. Appl. Phys. Lett..

[5] Sahoo K.C., Lin M.-K., Chang E.-Y., Lu Y.-Y., Chen C.-C., Huang J.-H., Chang C.-W. (2009). Fabrication of antireflective sub-wavelength structures on silicon nitride using nano cluster mask for solar cell application. Nanoscale Res. Lett..

[6] Lee Y.-J., Ruby D.S., Peters D.W., McKenzie B.B., Hsu J.W.P. (2008). ZnO nanostructures as efficient antireflection layers in solar cells. Nano Lett..

[7] Leem J.W., Kim S., Lee S.H., Rogers J.A., Kim E., Yu J.S. (2014). Efficiency enhancement of organic solar cells using hydrophobic antireflective inverted moth-eye nanopatterned PDMS films. Adv. Energy Mater..

[8] Rahman A., Ashraf A., Xin H., Tong X., Sutter P., Eisaman M.D., Black C.T. (2015). Sub-50-nm self-assembled nanotextures for enhanced broadband antireflection in silicon solar cells. Nat. Commun..

[9] Kwon Y.W., Park J., Kim T., Kang S.H., Kim H., Shin J., Jeon S., Hong S.W. (2016). Flexible near-field nanopatterning with ultrathin, conformal phase masks on nonplanar substrates for biomimetic hierarchical photonic structures. ACS Nano.

[10] Chang C.-C., Huang C.-H. (2022). ZnO Nanorods as Antireflection Layers in Metal-Insulator-Semiconductor Solar Cells. Electronics.

[11] Kim J.-H., Choi Y.J., Lee J., Lee S.G. (2022). Highly transparent antireflection coatings on fullerene-free organic solar cells using polymeric nanoparticles. Thin Solid Films.

[12] He Y., Zhang W., Zhang S., Kang X., Peng W., Xu Y. (2012). Study of the photoconductive ZnO UV detector based on the electrically floated nanowire array. Sens. Actuators A.

[13] Ho Y.-H., Ting K.-H., Chen K.-Y., Liu S.-W., Tian W.-C., Wei P.-K. (2013). Omnidirectional antireflection polymer films nanoimprinted by density-graded nanoporous silicon and image improvement in display panel. Opt. Express.

[14] Leem J.W., Song Y.M., Yu J.S. (2013). Biomimetic artificial Si compound eye surface structures with broadband and wide-angle antireflection properties for Si-based optoelectronic applications. Nanoscale.

[15] Kim J.K., Chhajed S., Schubert M.F., Schubert E.F., Fischer A.J., Crawford M.H., Cho J., Kim H., Sone C. (2008). Light-extraction enhancement of GaInN light-emitting diodes by graded-refractive-index indium tin oxide anti-reflection contact. Adv. Mater..

[16] Song Y.M., Choi E.S., Park G.C., Park C.Y., Jang S.J., Lee Y.T. (2010). Disordered antireflective nanostructures on GaN-based light emitting diodes using Ag nanoparticles for improved light extraction efficiency. Appl. Phys. Lett..

[17] Guo S., Zhou S., Li H., You B. (2015). Light diffusing films fabricated by strawberry-like PMMA/SiO_2_ composite microspheres for LED application. J. Colloid Interface Sci..

[18] Kanamori Y., Ishimori M., Hane K. (2002). High efficient light-emitting diodes with antireflection subwavelength gratings. IEEE Photonics Technol. Lett..

[19] Song Y.M., Choi E.S., Yu J.S., Lee Y.T. (2009). Light-extraction enhancement of red AlGaInP light-emitting diodes with antireflective subwavelength structures. Opt. Express.

[20] Wang Y., Zeng Z., Li J., Chi L., Guo X., Lu N. (2013). Biomimetic Antireflective Silicon Nanocones Array for Small Molecules Analysis. J. Am. Soc. Mass Spectrom..

[21] Chang H.-W., Lee J., Hofmann S., Kim Y.H., Müller-Meskamp L., Lüssem B., Wu C.-C., Leo K., Gather M.C. (2013). Nano-particle based scattering layers for optical efficiency enhancement of organic light-emitting diodes and organic solar cells. J. Appl. Phys..

[22] Shin C.-H., Shin E.Y., Kim M.-H., Lee J.-H., Choi Y. (2015). Nanoparticle scattering layer for improving light extraction efficiency of organic light emitting diodes. Opt. Express.

[23] Riedel D., Wehlus T., Reusch T.C.G., Brabec C.J. (2016). Polymer-based scattering layers for internal light extraction from organic light emitting diodes. Org. Electron..

[24] Chang C.-C., Lee K.-M., Huang C.-H. (2021). The Optical Properties of Metal-Free Polymer Films with Self-Assembled Nanoparticles. Polymers.

[25] Jenekhe S.A., Osaheni J.A. (1994). Excimers and exciplexes of conjugated polymers. Science.

[26] Zhang C., Zhang J., Zeng W., Zheng N., Li W., Gao W., Yu G., Yang C. (2016). Benzobisthiadiazole-*alt*-bithiazole copolymers with deep HOMO levels for good-performance field-effect transistors with air stability and a high on/off ratio. Polym. Chem..

[27] Ekimov A.I., Efros A.L., Onushchenko A.A. (1985). Quantum size effect in semiconductor microcrystals. Solid State Commun..

[28] Choi C.L., Koski K.J., Sivasankar S., Alivisatos A.P. (2009). Strain-dependent photoluminescence behavior of CdSe/CdS nanocrystals with spherical, linear, and branched topologies. Nano Lett..

[29] Zhong J., Yang X., Lou S., Zhou S. (2017). Substrate-dependent morphology and photoluminescence of MoS_2_ nanobelt arrays. Mater. Lett..

[30] Lippert S., Schneider L.M., Renaud D., Kang K.N., Ajayi O., Kuhnert J., Halbich M.-U., Abdulmunem O.M., Lin X., Hassoon K. (2017). Influence of the substrate material on the optical properties of tungsten diselenide monolayers. 2D Mater..

[31] Liu Z., Amani M., Najmaei S., Xu Q., Zou X., Zho W., Yu T., Qiu C., Birdwell A.G., Crowne F.J. (2014). Strain and structure heterogeneity in MoS_2_ atomic layers grown by chemical vapour deposition. Nat. Commun..

[32] Plechinger G., Schrettenbrunner F.-X., Eroms J., Weiss D., Schüller C., Korn T. (2012). Low-temperature photoluminescence of oxide-covered single-layer MoS_2_. Phys. Status Solidi RRL.

[33] Sercombe D., Schwarz S., Del Pozo-Zamudio O., Liu F., Robinson B., Chekhovich E., Tartakovski I., Kolosov O., Tartakovskii A. (2013). Optical investigation of the natural electron doping in thin MoS_2_ films deposited on dielectric substrates. Sci. Rep..

[34] Buscema M., Steele G.A., van der Zant H.S.J., Castellanos-Gomez A. (2014). The effect of the substrate on the Raman and photoluminescence emission of single-layer MoS_2_. Nano Res..

[35] Yu Y., Yu Y., Xu C., Cai Y.-Q., Su L., Zhang Y., Zhang Y.-W., Gundogdu K., Cao L. (2016). Engineering substrate interactions for high luminescence efficiency of transition-metal dichalcogenide monolayers. Adv. Funct. Mater..

[36] Yan R., Simpson J.R., Bertolazzi S., Brivio J., Watson M., Wu X., Kis A., Luo T., Hight Walker A.R., Xing H.G. (2014). Thermal conductivity of monolayer molybdenum disulfide obtained from temperature-dependent Raman spectroscopy. ACS Nano.

[37] Scheuschner N., Ochedowski O., Kaulitz A.-M., Gillen R., Schleberger M., Maultzsch J. (2014). Photoluminescence of freestanding single- and few-layer MoS_2_. Phys. Rev. B.

[38] Lin Y., Ling X., Yu L., Huang S., Hsu A.L., Lee Y.-H., Kong J., Dresselhaus M.S., Palacios T. (2014). Dielectric screening of excitons and trions in single-layer MoS_2_. Nano Lett..

[39] Keldysh L.V. (1980). Coulomb interaction in thin semiconductor and semimetal films. JETP Lett..

[40] Keldysh L.V. (1997). Excitons in semiconductor–dielectric nanostructures. Phys. Status Solidi A.

[41] Lee K.-M., Huang C.-H., Chang C.-Y., Chang C.-C. (2020). The optical and microstructural characterization of the polymeric thin films with self-assembly nanoparticles prepared by spin-coating techniques. Crystals.

[42] Islam K., Alnuaimi A., Battal E., Okyay A.K., Nayfeh A. (2014). Effect of gold nanoparticles size on light scattering for thin film amorphous-silicon solar cells. Solar Energy.

[43] Fu Y.H., Kuznetsov A.I., Miroshnichenko A.E., Yu Y.F., Luk’yanchuk B. (2013). Directional visible light scattering by silicon nanoparticles. Nat. Commun..

[44] Shi Y., Liu J., Yang Y. (2000). Device performance and polymer morphology in polymer light emitting diodes: The control of thin film morphology and device quantum efficiency. J. Appl. Phys..

[45] Liu J., Guo T.-F., Yang Y. (2002). Effects of thermal annealing on the performance of polymer light emitting diodes. J. Appl. Phys..

[46] Cury L.A., Guimarães P.S.S., Moreira R.L., Chacham H. (2004). Asymmetric line shape in the emission spectra of conjugated polymer thin films: An experimental signature of one-dimensional electronic states. J. Chem. Phys..

[47] Shin M., Kim H., Park J., Nam S., Heo K., Ree M., Ha C.-S., Kim Y. (2010). Abrupt Morphology Change upon Thermal Annealing in Poly(3-Hexylthiophene)/Soluble Fullerene Blend Films for Polymer Solar Cells. Adv. Funct. Mater..

