# Supplementary material for: The Characteristics of the Metal-Free and Non-Conjugated Polymer Film with Self-Assembled Nanoparticles
Source: Nanomaterials (Basel). 2023 Feb 2;13(3):596. doi: 10.3390/nano13030596 (PMC9921736; doi:10.3390/nano13030596)
Supplement: Supplementary file 1 [file nanomaterials-13-00596-s001.zip › nanomaterials-2147252-supplementary.pdf]

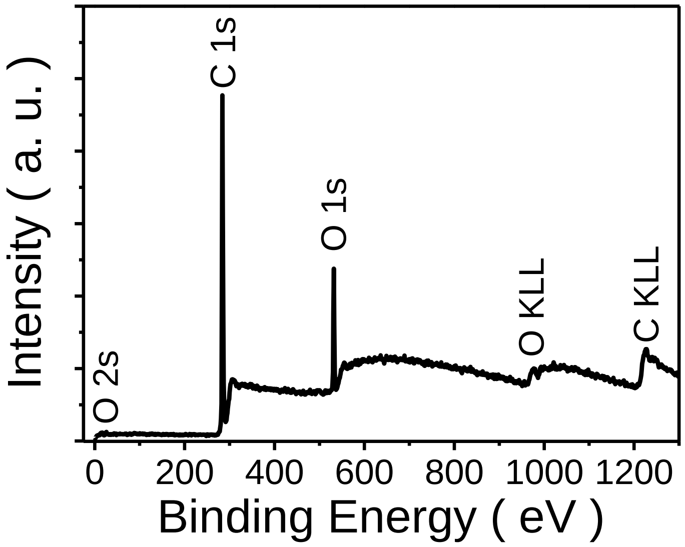

Figure S1. XPS spectrum of MP1D film was recorded on an X-ray photoelectron spectrometer (ULVAC-PHI, PHI Quantera II, Kanagawa, Japan).

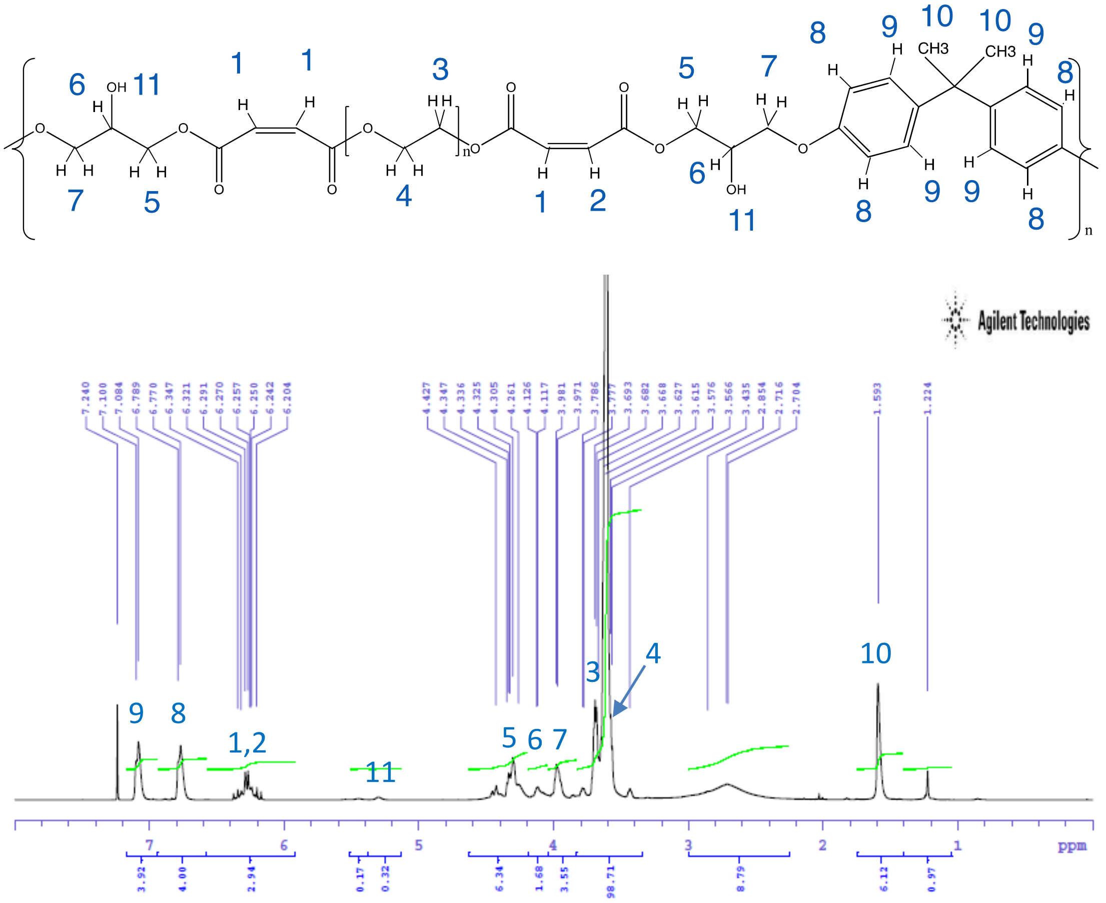

Figure S2. Chemical structure and the ^{1}H NMR (400 MHz, $CDCl_3$) spectrum of MP1D polymer.

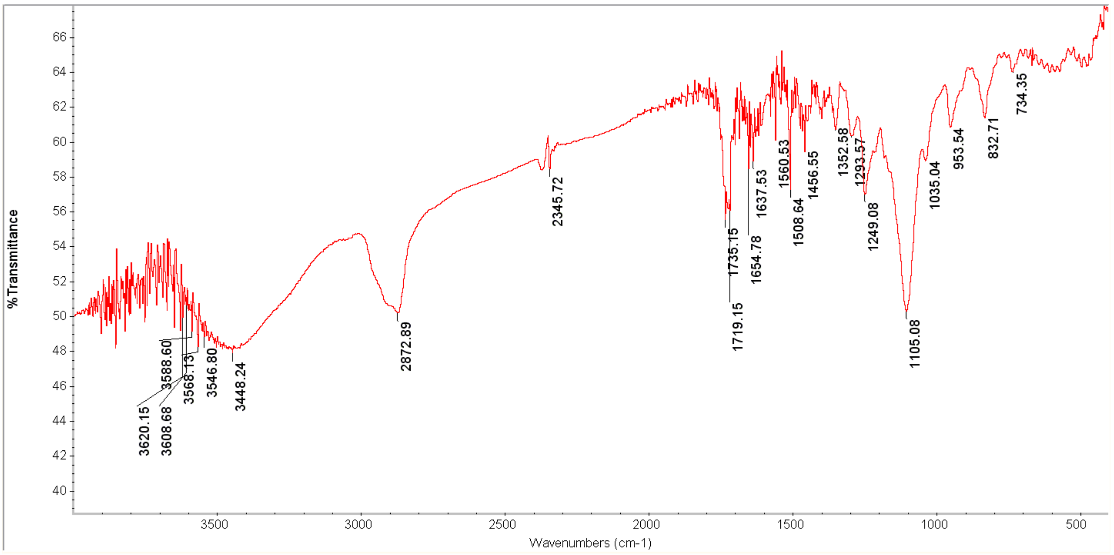

Figure S3. IR spectrum of MP1D polymer was recorded on a model 5700 spectrometer (Nicolet, Madison, SD, USA). The spectrum shows a strong broad band between 3200 cm^{-1} and 3600 cm−1 which is the characteristic OH group stretching frequency. The sharp band at 1700–1740 cm^{-1} is due to the stretching frequency of the ester carbonyl group. The bands at 2850-2960 cm^{-1} may stand for the stretching frequency of the CH, CH_2 and CH_3 groups. The band at around 1640 cm^{-1} is the characteristics bisphenol ring C=C and maleate HC=CH stretching. The bands at 734 and 833 cm^{-1} are due to the bending frequency of the bisphenol ring CH. The band at 954 cm^{-1} is the characteristics trans olefinic C–H bending.

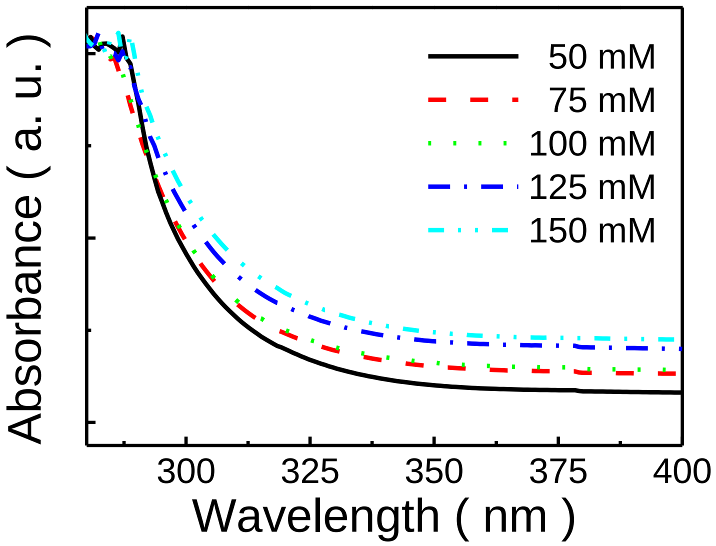

Figure S4. UV-Vis absorption spectra of MP1D films coated from various solutions (50, 75, 100, 125 and

150 mM). The optical band-gap energy of MP1D is around 4.05 eV.

The optical absorption edge can be used to estimate the optical band-gap energy (E_g). An exciton is created when a photon is absorbed. Accordingly, the optical E_g is obtained from the onset of the absorption spectrum. The absorption onset is derived from linear extrapolation of the low energy absorption edge. Hence, when solution concentrations are 50, 75, 100, 125 and 150 mM, the onset wavelengths (λ_{onset}) are 303.1, 303.6, 306.6 nm, 307.4 and 307.6 nm, respectively. The optical E_g is determined by:

$$E_g = 1240/\lambda_{onset},$$

where λ_{onset} is the onset wavelength. As a result, the optical E_g of MP1D film decreases from 4.09 to 4.03 eV when the concentration of the solution rises from 50 to 150 mM.

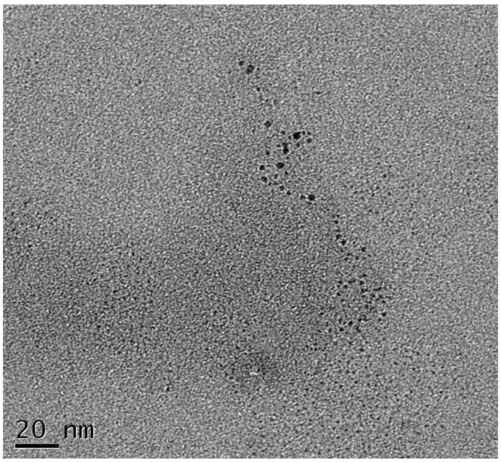

(**a**)

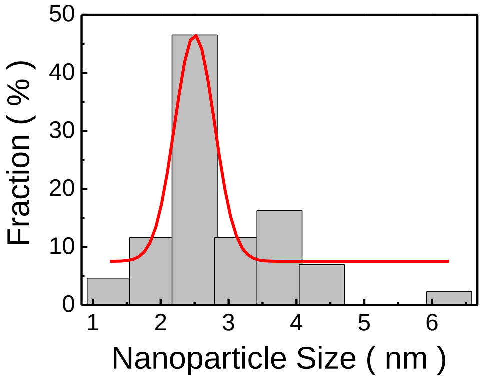

(**b**)

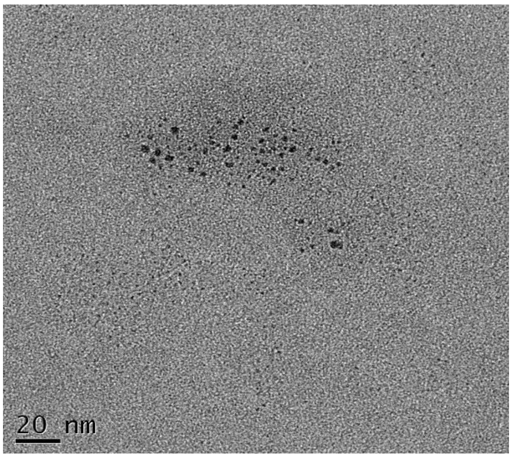

(**c**)

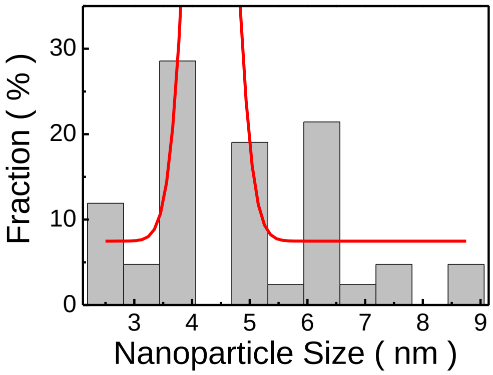

(**d**)

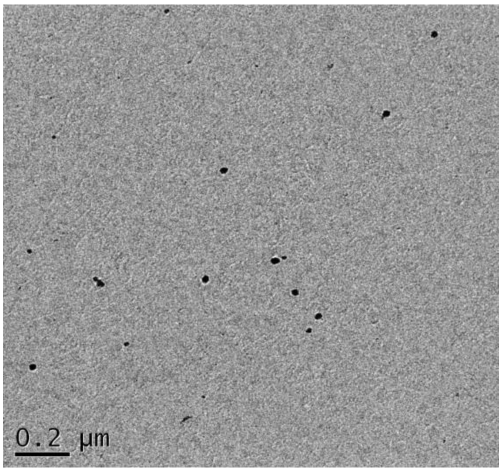

(e)

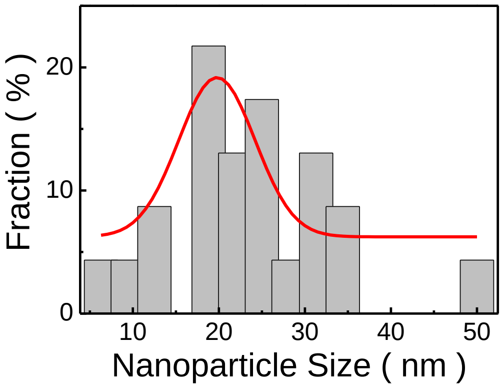

(f)

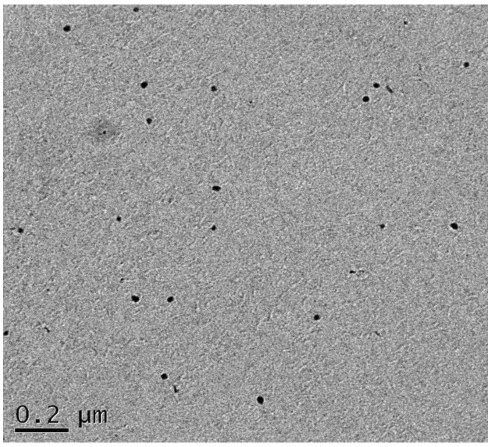

(g)

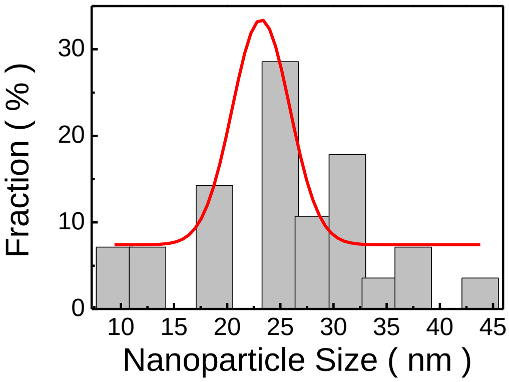

(h)

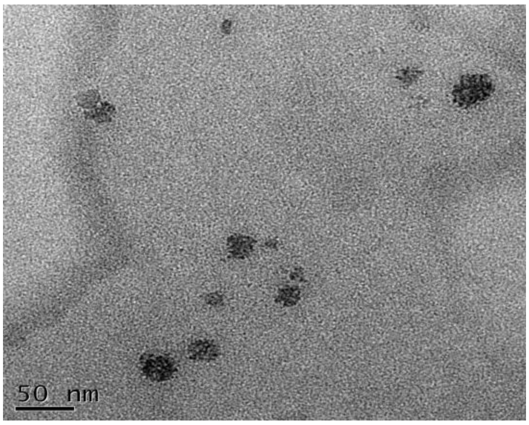

(i)

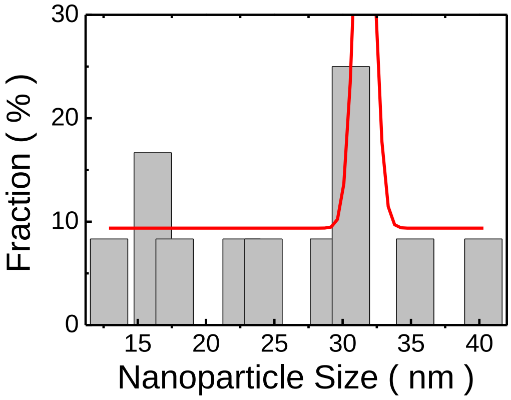

(j)

Figure S5. The TEM images of MP1D films prepared from different MP1D solutions: (**a**) 50 mM; (**c**) 75 mM; (e) 100 mM; (g) 125 mM; (i) 150 mM. The histogram of the size distribution of self-assembled

nanoparticles embedded in MP1D films prepared from different MP1D solutions: (**b**) 50 mM; (**d**) 75 mM; (f) 100 mM; (h) 125 mM; (j) 150 mM. (JEOL JEM-2100 TEM, Tokyo, Japan)
